# The Role of Power Doppler Ultrasonography in Caudal Epidural Injection

**DOI:** 10.3390/medicina58050575

**Published:** 2022-04-22

**Authors:** Yueh-Hsun Tsai, Guo-Shu Huang, Chi-Tun Tang, Fu-Chi Yang, Yi-Chih Hsu

**Affiliations:** 1 Department of Surgery, Tri-Service General Hospital, National Defense Medical Center, Taipei 11490, Taiwan; drxelamai@gmail.com; 2 Department of Radiology, Tri-Service General Hospital, National Defense Medical Center, Taipei 11490, Taiwan; gshuang5@gmail.com; 3 Department of Medical Research, Tri-Service General Hospital, National Defense Medical Center, Taipei 11490, Taiwan; 4 Department of Neurosurgery, Tri-Service General Hospital, National Defense Medical Center, Taipei 11490, Taiwan; omeprazon8@hotmail.com; 5 Department of Neurology, Tri-Service General Hospital, National Defense Medical Center, Taipei 11490, Taiwan; fuji-yang@yahoo.com.tw

**Keywords:** epidural injections, interventional ultrasonography, Doppler ultrasonography, doppler ultrasounds, power Doppler ultrasonography, caudal epidural injection, ultrasound-guided epidural injection, doppler ultrasound imaging

## Abstract

*Background and Objectives*: Although the ultrasound-guided technique is used in caudal epidural injections, severe complications can happen if ultrasound cannot identify the occurrence of intravascular injection. To determine intraepidural and intravascular injection during caudal epidural injections, we used power Doppler ultrasonography (PDU) when injecting medications into the epidural space. *Materials and Methods*: This is a retrospective study that enrolled a total of 277 patients with refractory low back pain or degenerative disc from January 2019 to December 2019. The injectate flow of caudal epidural injections was examined with the assistance of PDU and confirmed by fluoroscopy. Four flow patterns were identified by PDU in our study: the “Earthworm sign,” the “Patch sign,” the ”Tubular sign” and the “Absent flow sign.” The accuracy of PDU in identifying intraepidural and intravascular injections was determined by fluoroscopy images recorded during each injection. *Results*: We evaluated 277 patients (mean age, 68.6 ± 13.2 years; 106 men). The “Patch sign” showed a sensitivity of 88.76% and a specificity of 80% in predicting epidural injection without intravascular injection. The “Earthworm sign” demonstrated a sensitivity of 70% and a specificity of 100% in detecting intravascular injection. The “Tubular sign” showed a specificity of 100% and a sensitivity of 9.4% in predicting successful epidural injection. The absence of a flow signal showed a sensitivity of 1.87% and a specificity of 90% in predicting successful epidural injection. *Conclusions*: Ultrasound-guided caudal epidural injection can accurately determine intraepidural and intravascular injections with the assistance of PDU and is thus a good alternative technique to fluoroscopy-guided caudal epidural injection.

## 1. Introduction

Low back pain is a common medical problem that affects patients worldwide. Epidural steroid injection is an alternative treatment for refractory low back pain with degenerative disc disease and disc herniation that do not respond to analgesics, anti-inflammatory drugs, and physical therapy [1]. Corticosteroids can achieve high local concentrations by direct injection into the epidural space, reducing local inflammation by inhibiting pro-inflammatory substances [2]. Epidural steroid injections can be performed via caudal, interlaminar, or transforaminal approaches, depending on the injection route. A recent meta-analysis demonstrated that different approaches to epidural steroid injections exhibited similar effectiveness in managing radicular pain [3].

Caudal epidural injection (CEI) is relatively easy to achieve via the sacral hiatus. It also involves a minimal risk of accidental dural puncture [4]. Despite the relatively low operating risk compared with other approaches, CEI still has some rare complications. It has been reported that serious complications such as spinal cord infarction, subdural hematoma, and cerebellar infarction can occur following inadvertent intravascular injection [5,6]. Hence, studies suggest that epidural injection should proceed under fluoroscopic guidance with a contrast medium [7].

Ultrasonography (US) is a relatively cheap, easily accessible, and radiation-free tool that does not require contrast media, unlike fluoroscopy. Several studies discussing the effectiveness of US-guided and fluoroscopy-guided CEIs have been conducted, wherein US-guided CEI showed outcomes of pain reduction and success rate similar to those of fluoroscopy-guided injection [4,8,9]. Nevertheless, a main limitation of US-guided CEI is that it cannot precisely monitor intraepidural and intravascular injections. Although the “blood flashback” test is applied during CEI to detect intravascular injection, a study showed that it only has a sensitivity of 44.7% [10]. Meanwhile, inadvertent intravascular injection has been detected under fluoroscopy in 8% of CEI cases [11,12]. Several studies have described the appearance of Color Doppler US (CDU) during intraepidural fluid injection in US-guided CEI [9,13]; nevertheless, intravascular injection was not detectable. Serious complications such as spinal cord infarction, subdural hematoma, cerebellar infarction, and death may occur if intravascular injection cannot be discovered [14,15]. Consequently, it is important to detect intravascular injection in US-guided CEI.

Power Doppler US (PDU) has greater sensitivity than CDU for detecting and demonstrating blood flow in small vessels [16,17]. Moreover, PDU is not prone to aliasing and is less dependent on angular variations [18]. It also presents a better resolution of intravascular imaging and constancy of flow than CDU [17]. In our study, we used PDU to distinguish the flow signals of intraepidural and intravascular injections during US-guided CEI. This study aimed to determine the feasibility of using PDU to monitor intraepidural and intravascular injections during US-guided CEI.

## 2. Materials and Methods

### 2.1. Patients

This was a retrospective study involving patients with refractory low back pain, spinal stenosis, or degenerative disc, indicated to receive CEI from January 2019 to December 2019. Standardized CEI techniques have been performed in our institution since 2019. We replaced CDU with PDU, which is more sensitive for detecting vascular flow [16]. Exclusion criteria included pregnancy, psychiatric disorders, bleeding tendency, signs of infection, and inflammatory disease. Patients who could not tolerate the procedure, were allergic to the contrast media, or presented difficulties in visualizing the needle tip in the epidural space were also excluded from this study. Approval from the Institutional Review Board of Tri-Service General Hospital, National Defense Medical Center was obtained, (IRB Number: B202105046), which included a waiver of informed consent owing to the study’s retrospective nature.

### 2.2. Procedures

**Figure 1:**
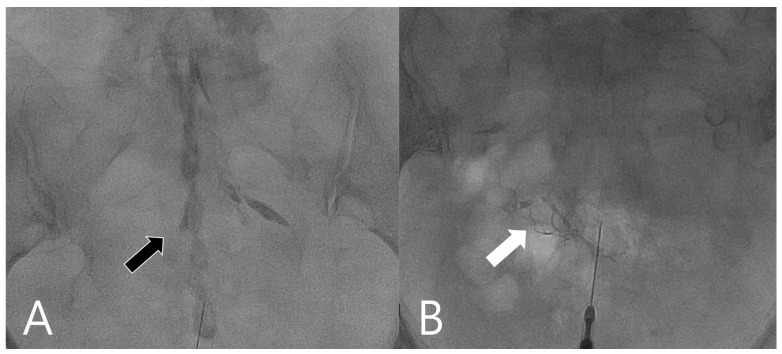
Fluoroscopy image of intraepidural and intravascular injection. (**A**). Intraepidural injection shows contrast in the epidural space (black arrow) with Christmas tree appearance. (**B**). Intravascular injection shows contrast in small arteries (white arrow) with early contrast washout.

The CEI techniques were performed following the standardized protocol previously described by Yoon et al. [9]. All procedures were performed by an interventional radiologist (Y.-C.H.) with more than 10 years of experience in musculoskeletal US and epidural injection. US was performed using a scanner (Xario 100; Toshiba, Tokyo, Japan) with a 7–18 MHz linear transducer or a 1–6 MHz curve transducer in obese patients. In order to avoid intravascular injection, which may cause devastating complication such as paraplegia, standardized US-guided and fluoroscopy-assisted techniques have been routinely included in our protocol since 2015 [19]. The ultrasound machine was routinely arranged next to a digital angiography system (ARTIS Q; SIEMENS, Munich, Germany). These patients were prepared using a hygienic technique in the prone position. First, we palpated the sacral hiatus. We transversely placed the US probe in the center to identify the sacral hiatus. At this stage, the ultrasound transducer was rotated 90° to obtain the longitudinal view of the sacral hiatus. A 23G injection needle was inserted into the sacral hiatus. The loss-of-resistance technique was utilized while penetrating the sacrococcygeal ligament and seating the needle tip in the epidural space. After the needle in the epidural space was confirmed on gray-scale US, we verified the absence of blood in the syringe and applied negative pressure to ensure that no blood or cerebrospinal fluid was aspirated. If blood appeared in the needle hub, it was removed, and the needle was repositioned in a different site. A test dose of 0.5 mL of 5% dextrose in water was injected under PDU based on a previous study published in 2019 [20]. The radiographer next to the interventional radiologist was asked to initiate ultrasound recording when the needle was inserted into the sacral canal and when the test solution was injected. Two 5 s videos were captured and saved into a picture archiving and communication system (PACS). Then, the operator captured the image of maximal power doppler amplitudes in the canal, which were saved into the PACS. Furthermore, intra-epidural injection or intravascular injection was confirmed via fluoroscopy with contrast medium (Omnipaque 300 mg/mL; 1–2 mL) injection [21]. (Figure 1) Once the proper position was confirmed, 7.5 mL of solution (2.5 mL 10 mg/L triamcinolone and 5 mL of water) or 7.5 mL of 5% dextrose was used for epidural injection.

### 2.3. Data Collection and Analysis

**Figure 2:**
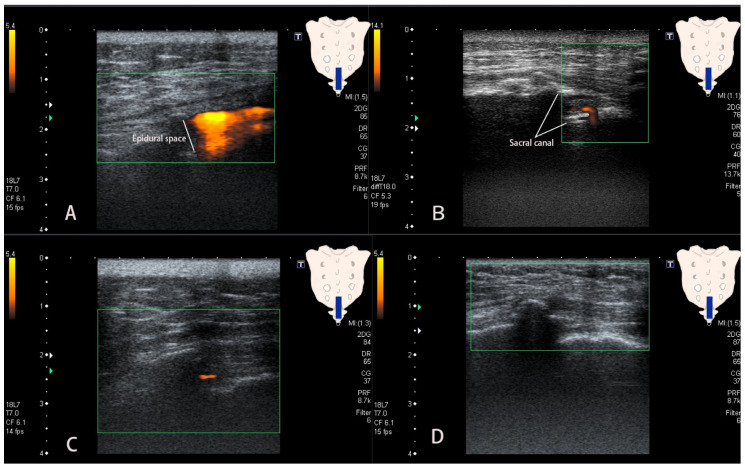
The flow signal in Power Doppler Ultrasound images. (**A**). Patch sign: flow filling the epidural space. (**B**). Earthworm sign: signal comprising a tubular flow descending through the sacral canal floor. (**C**). Tubular sign: signal in the sacral canal that did not fill more than half of its diameters. (**D**). Absent flow sign: no flow observed.

Clinical data including sex, age, history of laminectomy, and body mass index (BMI) were routinely recorded. All US and fluoroscopy images were saved in the PACS. Four different flow patterns were observed under PDU. The first pattern was defined as a patch sign consisting of an affluent flow filling the epidural space. We defined the patch sign as positive when the flow filled more than half of the sacral canal diameter (Figure 2A). The second pattern was defined as “earthworm”-shape flow and consisted of a tubular flow occurring near the deep level of the sacral canal descending through the bony floor of the same canal (Figure 2B). The third pattern was a tubular sign, which appeared in the sacral canal without filling more than half of the sacral canal diameters (Figure 2C). The fourth pattern presented the absence of a flow image under PDU (Figure 2D). The PDU and fluoroscopy images were analyzed independently by two readers who were blinded to the results of the clinical data. The two readers reached a consensus on the pattern of the PDU images. The results were compared with the outcomes (intraepidural or intravascular injection) confirmed under fluoroscopy.

### 2.4. Statistics Analysis

Statistical evaluation was performed using the *t*-test (age, BMI) and Fisher’s exact test (sex, history of laminectomy) (SPSS Version 25). The interobserver agreement of the PDU flow pattern was assessed using Cohen’s kappa statistics. The kappa value was interpreted as follows: 0.41–0.60, moderate; 0.61–0.80, substantial; and 0.81–1.00, almost perfect agreement [22]. The PDU images of flow patterns during US-guided injections were compared with the fluoroscopy results of intraepidural or intravascular injection. Sensitivity, specificity, positive likelihood ratio (PLR), and negative likelihood ratio (NLR) were calculated for each PDU flow pattern. Data were interpreted using McGee’s scale to determine the likelihood ratio according to Ref. [23], with slight adjustments for a better understanding: 1. Evidence to rule in disease: PLR > 10, strong; 5–10, moderate; 2–5, fair; and >1, low. 2. No diagnostic value: PLR = 1. 3. Evidence to rule out disease: PLR of 0.5~0.9, weak; 0.2~0.5, fair; 0.1~0.2, moderate; and <0.1, strong.

## 3. Results

**Figure 3:**
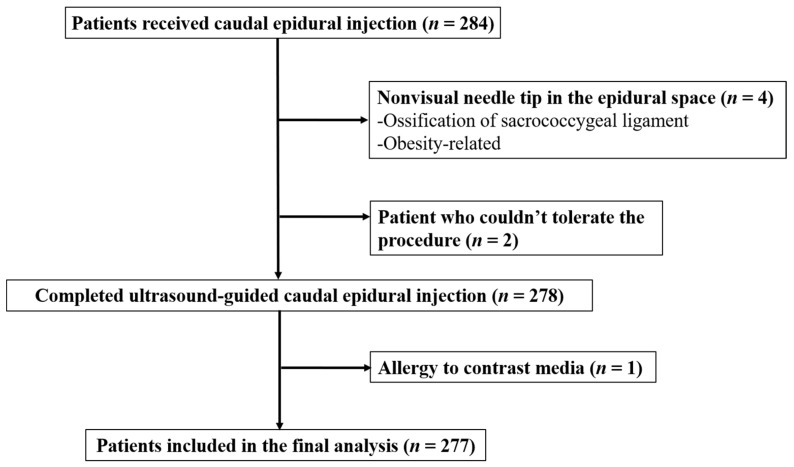
Flow diagram for patient selection.

From January 2019 to December 2019, 284 patients received US-guided CEI. Patients were excluded if visualization of the needle tip in the epidural space under ultrasonography was not possible or if contrast fluoroscopy was contraindicated. Four patients were excluded due to the inability to visualize the needle tip in the epidural space (ossification of the sacrococcygeal ligament, obesity-related), one could not undergo fluoroscopy because of an allergy to the contrast medium, and two could not undergo the US-guided procedure due to severe back pain. A total of 277 patients were included in the study. A complete patient selection flow diagram is shown in Figure 3.

There were 171 women and 106 men with a mean age of 68.6 ± 13.2 years (range, 23–97 years). Mean BMI was 24.7 ± 4.0 kg/cm^2^ (range, 16.9–44.7 kg/cm^2^). Seventy-four patients (26.7%) had a history of laminectomy. The patients involved in the study were able to complete the procedure without any severe side effects. Inadvertent intravascular injections occurred in 10 patients (3.61%) with fluoroscopic confirmation. Needle replacement was performed again under fluoroscopy, with the outcome of proper intraepidural injection. There was no significant difference (*p* > 0.05) in the incidence of inadvertent intravascular injections in patients based on sex, age, BMI, and history of laminectomy (Table 1).

The PDU flow results observed by both researchers were concordant as regards absent flow sign and earthworm sign. Discordance happened when the second reviewer identified 10 cases of tubular sign as patch sign. The kappa coefficient for interobserver agreement was 0.835, indicating a very high agreement. The results revealed 239 cases with patch signs, 7 cases with earthworm signs, 25 cases with tubular signs, and 6 cases without flow on PDU (Table 2). The sensitivity, specificity, PLR, and NLR for each flow pattern under PDU to predict intraepidural injection in CEI are shown in Table 3. Table 4 discusses the accuracy of each pattern in predicting intravascular injection in the CEI. When patch signs were found on PDU, the sensitivity, specificity, PLR, and NLR when determining intraepidural injection were 88.8%, 80.0%, 4.44, and 0.14, respectively, while they were 20%, 11.2%, 0.22, and 7.12, respectively, when determining intravascular injection. When earthworm signs were found on PDU, the sensitivity, specificity, PLR, and NLR for intraepidural injection were 0%, 30.0%, 0%, and 3.3%, respectively, while they were 70%, 100%, infinity, and 0.3, respectively, for intravascular injection. When tubular signs were found on PDU, the sensitivity, specificity, PLR, and NLR for intraepidural injection were 9.4%, 100%, infinity, 0.91, respectively, while they were 0, 90.6%, 0, and 1.1, respectively, for intravascular injection. When no flow was observed on PDU, the sensitivity, specificity, PLR, and NLR for intraepidural injection were 1.87%, 90%, 0.91, 1.09, respectively, while they were 10%, 98.1%, 5.34, and 0.92, respectively, for intravascular injection.

## 4. Discussion

We investigated the feasibility of PDU assistance for US-guided CEI. To detect intraepidural and intravascular injections during CEI, we used PDU to identify the flow signal and classified the signal into four flow patterns, with high interobserver agreement. We found that the “Patch sign” was detected in 88.76% of successful epidural injections; the “Earthworm sign” showed 100% specificity for intravascular injection. This is the first study applying US to detect both intraepidural and intravascular injections during CEI.

In our study, the patch signal under PDU was a positive sign of intraepidural injection during CEI. The patch sign showed high specificity (88.76%) and sensitivity (80%) for predicting intraepidural injection with moderate evidence (PLR: 4.44). When fluid is injected into the epidural space, it creates a free flow filling the sacral canal [9,13], which presents a patch-pattern flow on PDU images. Two of the 239 cases with a “Patch sign” presented intravascular injections. Nevertheless, in very few cases, Doppler artifacts can be caused by the motion of the transducer or of objects. The flash artifact is an abrupt appearance of colored flow that fills the image, most commonly seen in hypoechoic areas [18].

The “Earthworm sign” appeared to be a good diagnostic sign to detect intravascular injection in CEI because of the very high PLR ratio. It also showed ahigh specificity (100%) and sensitivity (70%) when determining intravascular injection. Previous studies of CDU-assisted US-guided CEI were not able to detect intravascular injection [9,13,21]. The results of our study indicate a progress in detecting intraepidural and intravascular injection thanks to the advantages of PDU that allows better resolution of intravascular imaging and constancy of flow than CDU [16,17]. The distribution of the epidural vasculature concentrated in the front wall of the sacral canal and passing through the vertebral bodies [24] is probably the reason behind the “Earthworm sign” identified by PDU in the case of intravascular injection. If the needle is located in the anterior vessel-rich zone of the sacral canal, causing intravascular injection, the solution may flow into the vertebral bodies through the vessels, resulting in an earthworm-shaped tubular flow signal descending through the floor of the sacral canal. Although devastating complications occur if arterial steroid injection is performed rather than intravenous injection, caudal epidural injection is safe as long as it is possible to detect any vascular injection and move the needle tip to the epidural space. We speculate that intracanal pressure was elevated during CEI. Jessica et al. reported that a transient and obvious increase in pressure in the epidural space occurred following a solution administration [25]. We found that 25 cases exhibited tubular flow and 5 cases exhibited no flow signal on PDU during intraepidural injection; one case, i.e., the patient with intravascular injection, revealed an absent flow signal on PDU.

We also analyzed some of the factors that may be related to a higher risk of inadvertent intravascular injection. In some previous studies, age was correlated with a higher incidence of intravascular injection [26,27]. However, our study revealed no significant differences in age, sex, BMI, or laminectomy history, corroborating the results reported by Nikooseresht et al. [28] which showed that anatomic variation is not predicted by age, sex, BMI, or history of laminectomy.

Several studies have been conducted using US-guided CEI [4,8,13,21,28,29], but no study has determined intraepidural and intravascular injections during CEI. However, the “blood flashback” test in US-guided CEI was not sufficiently sensitive, as indicated by a previous study that revealed that this test that looks for a flashback of blood only has a sensitivity of 44.7% [10]. There are several reasons why less than half of the intravascular injections were predicted by the blood flashback test. First, the negative pressure applied during aspiration may collapse the blood vessels, thereby stopping the flashback [10]. Second, the repositioning of the needle after noticing a flashback can also result in blood clotting in the needle hub, which prevents blood flashback during reinjection. Hence, we performed US-guided CEI with the assistance of PDU and successfully detected intraepidural and intravascular injections.

This study has several limitations. First, this was a retrospective study. However, it is difficult to perform a double-blind control trial without a traditional method, such as fluoroscopy, before ensuring the feasibility of US-guided CESI. Second, our study only involved cases allowing a clear visualization of the needle tip in the epidural space. Bony artifacts, ossification of the sacrococcygeal ligament, or obesity could cause difficulties in visualizing the needle tip in the epidural space [29]. In such patients, more studies should be conducted to prove the feasibility of PDU-assisted CEI. Third, this study was conducted only on CEI; however, ultrasonography has been useful for other epidural injection techniques, such as transforaminal injection [30,31,32]. The application of PDU during transforaminal epidural injection requires more large-scale and cautious studies to prove its feasibility and safety.

## 5. Conclusions

In conclusion, US-guided CEI can determine intraepidural and intravascular injections with the assistance of PDU and is a good alternative technique to fluoroscopy-guided CEI.

## Figures and Tables

**Table 1:** Demographic data.

Variable	Intravascular Injection	Intraepidural Injection	*p*-Value
Sex			0.327
male *	2	104	
female *	8	163	
Age (y)			0.155
range	56~86	23~97	
mean ± SD	74.4 ± 9.3	68.4 ± 13.3	
BMI (kg/cm^2^)			0.347
range	16.9~28.0	16.9~44.7	
mean ± SD	23.5 ± 4.0	24.7 ± 3.9	
History of laminectomy			0.138
yes *	5	69	
no *	5	198	

* Data indicate the number of patients. SD: standard deviation.

**Table 2:** Incidence of intravascular caudal epidural injection by flow pattern under Power Doppler ultrasonography.

Variable	Intravascular Injection	Intraepidural Injection	*p*-Value
Flow pattern			<0.001
Patch sign	2	237	
Earthworm sign	7	0	
Tubular sign	0	25	
Absent flow	1	5	

**Table 3:** Diagnostic performance of each flow pattern under Power Doppler ultrasound in predicting intraepidural injection during caudal epidural injection.

	Sensitivity% [95% CI]	Specificity% [95% CI]	PLR [95% CI]	NLR [95% CI]
Flow pattern				
Patch sign	88.8 [84.35–92.29]	80.0 [44.39–97.48]	4.44 [1.28–15.34]	0.14 [1.28–15.34]
Earthworm sign	0 [0–1.37]	30 [6.67–65.25]	0	3.3 [1.29–8.59]
Tubular sign	9.4 [6.15–13.51]	100 [69.15–100]	∞	0.91 [0.87–0.94]
Absent flow	1.87 [0.61–4.32]	90.0 [55.50–99.75]	0.19 [0.02–1.46]	1.09 [0.89–1.34]

PLR: positive likelihood ratio. NLR: negative likelihood ratio.

**Table 4:** Diagnostic performance of each flow pattern under Power Doppler ultrasound in predicting intravascular injection during caudal epidural injection.

	Sensitivity% [95% CI]	Specificity% [95% CI]	PLR [95% CI]	NLR [95% CI]
Flow pattern				
Patch sign	20 [2.52–55.61]	11.2 [7.71–15.65]	0.22 [0.07–0.78]	7.12 [4.50–11.26]
Earthworm sign	70 [34.75–93.33]	100 [98.63–100]	∞	0.3 [0.12–0.77]
Tubular sign	0 [0–30.85]	90.6 [86.49–93.85]	0	1.1 [1.06–1.15]
Absent flow	10 [0.25–44.5]	98.1 [95.68–99.39]	5.34 [0.69–41.57]	0.92 [0.75–1.13]

PLR: positive likelihood ratio. NLR: negative likelihood ratio.

## Data Availability

Not applicable.
